# Monitoring of incidence, severity, and causality of adverse drug reactions in hospitalized patients with cardiovascular disease

**DOI:** 10.4103/0253-7613.75661

**Published:** 2011-02

**Authors:** Sharminder Kaur, Vinod Kapoor, Rajiv Mahajan, Mohan Lal, Seema Gupta

**Affiliations:** Department of Pharmacology, Government Medical College, Jammu - 180 001, India; 1 Department of Pharmacology, Adesh Institute of Medical Sciences and Research, Bathinda - 151 109, India; 2 Department of Cardiology, Government Medical College, Jammu - 180 001, India

**Keywords:** Adverse drug reaction, cardiology, causality, monitoring

## Abstract

**Background:**

Patients admitted to cardiology department are mostly on polypharmacy. So drug-drug interactions and adverse effects of drugs are quite common. Yet, there is a paucity of data regarding adverse drug reaction (ADR) monitoring in cardiology department in India. The present study is an effort to fill up these lacunae.

**Materials and Methods:**

A prospective, observational study registering 966 indoor cardiology patients according to predetermined inclusion and exclusion criteria was conducted for one year. ADR profile was noted by spontaneous reporting and intensive monitoring. Naranjo ADR probability scale was used to establish the causality.

**Results:**

A total of 208 ADRs were reported from 188 patients (19.5%). Of these 188 patients, 62 patients (33%) were hospitalized primarily due to the development of ADRs, while 126 (67%) patients developed ADRs during hospital stay. Nitrates were the most common offender drug group (17.8%).

**Conclusion:**

Development of ADR in one of every five cardiac patient points toward a grave situation, but a higher incidence of Type A reactions in cardiology department means that these can be avoided.

## Introduction

With the addition of a multitude of drugs to the physician’s armamentarium, the treatment of many hitherto uncontrollable diseases has become possible. However, every progress has a price to pay, so is the case with the new drugs which have led to the iatrogenic diseases. Although most patients derive far more benefit than harm, a proportion of them experience adverse drug reactions (ADRs) from the use of the medicines at recommended doses and frequencies.

It is estimated that ADRs are responsible for approximately 5% of hospital admissions. Epidemiological research performed in the United States shows the occurrence of ADRs in 10 to 20% of all hospital inpatients. These are estimated to cause death in 0.1% of medical and 0.01% of surgical inpatients.[1]

Cardiovascular diseases (CVDs) remain a leading cause of morbidity and mortality worldwide. More than 30% of all the deaths every year are attributed to CVDs.[2] With such a sizeable number of people suffering from these diseases, the number of drugs consumed per capita is quite predictable and thus the inevitable danger of ADRs. Cardiovascular medications have been cited as one of the most common class of drugs associated with medication errors and ADRs, which need to be monitored from time to time.[3] The adverse drug event (ADE) prevention study group reported that odds ratio (OR) of severe ADEs with cardiovascular medication was 2.4 times that of other medications.[4]

However, sufficient data pertaining to ADRs in Indian population has still not been generated. Pharmacovigilance has not picked up well in India and the subject is in its infancy. India rates below 1% in pharmacovigilance as against the world rate of 5%. This is due to ignorance of the subject and also lack of training.[5] India is the fourth largest producer of pharmaceuticals in the world. India is a vast country with more than 6 000 licensed drug manufacturers and more than 60 000 branded formulations. It is also emerging as a clinical trials hub. Many new drugs are being introduced in the country, so there is an immense need to improve the pharmacovigilance system to protect the Indian population from potential harm that may be caused by some of the new drugs.[6]

We are heavily dependent on the data generated in other countries and advisory notes issued and regulatory actions taken by regulators elsewhere, making it imperative upon us to generate Indian data. There is no such pharmacovigilance system working presently in our set-up and moreover, there is paucity of data regarding ADR monitoring, especially in relation to drugs used for the management of various cardiovascular conditions in India as a whole. Accordingly, the present study was designed to monitor the incidence, severity, and causality of ADRs in indoor cardiology patients to fill up these lacunae.

## Materials and Methods

It was a prospective, observational study, commenced after approval from Institutional Ethical Committee and was spread over a period of one year. Indoor cardiac patients were registered in the study, according to certain inclusion and exclusion criteria and after taking informed written consent.

### 

#### Inclusion criteria

These included the following:

1. Adult patients of both gender admitted in the cardiology department receiving various drugs.
2. Both new and already admitted patients at the time of commencement of study.

#### Exclusion criteria

1. Patients less than 18 years of age.
2. Cases of therapeutic failures; intentional or accidental poisoning; with a history of drug abuse and of noncompliance.
3. Pregnant and lactating females.
4. Patients not perceiving the questionnaire.

A total of 1024 patients were enrolled in the study, of which 966 patients were finally registered. Two methods, spontaneous reporting and intensive monitoring, were employed to assess the ADR profile in the patients.

For spontaneous monitoring, treating physicians were provided with reporting cards on which they recorded suspected ADRs. After the initial notification of the suspected ADR by the physician, additional detail was collected by review of the patient case records and interviews. The collected data were further analyzed and causality was ascertained.[7]

In the second method, that is, intensive monitoring which is based upon systematic evaluation of link between the adverse event and drugs involved, patients admitted to cardiology unit were followed up until discharged. Of these patients, cases of suspected ADRs were enrolled separately. For the information which was not recorded in the medical case sheets, direct patient interviews were conducted using a structured questionnaire.[8] The information was transferred to the performa which was adopted from the one used by Indian Council of Medical Research,[9] after modifying it according to the study requirements.

ADRs were characterized using Rawlins and Thompson classification.[10] They were categorized into mild, moderate, and severe ADRs, according to severity scale.[11] The causality relationship between the drug and the effect was established using Naranjo’s ADR probability scale.[12]

#### Statistical Analysis

Statistical analysis was performed by using Microsoft Excel and SPSS 10.0.1 for windows. Univariate analysis was carried out using Chi Square test and Z test for proportions. Odd Ratio(OR) with 95% confidence interval (CI) was calculated for assessing risk of each variable with the outcome, that is, occurrence of ADR. Multivariate analysis was performed to assess the independent risk of variables found significantly on univariate analysis by performing a stepwise logistic regression analysis. A *P* value of <0.05 was considered significant unless specified otherwise.

## Results

The age of patients enrolled in the study varied from 25 to 95 years, with a mean age of 61 ± 13.47 years. Of 966 patients enrolled, 354 (36.3%) patients were aged above 65 years. A total of 672 (69.5%) males and 294 (30.5%) females were included in the study [Table 1].

**Table 1:** Characteristics of all enrolled patients and patients with ADRs

*Characteristic*	*Number of patients (%)(n = 966)*	*Patients with ADR (%)(n = 188)*	*Odd ratio (OR)*	*95% CI*
Sex				
Male	672 (69.5)	106 (15.8)	2.07	1.47–2.91
Female	294 (30.4)	82 (27.9)		
Age (years)				
≤65	612 (63.3)	100 (16.3)	1.5	1.08–2.09
>65	354 (36.6)	88 (24.9)		
Comorbid condition				
≤2	655 (67.8)	70 (10.7)	6.16	4.30–8.83
>2	311 (32.1)	118 (37.9)		
Number of drugs				
≤10	261 (27.1)	22 (8.4)	5.21	3.17–7.63
>10	705 (72.9)	166 (23.5)		

ADR = adverse drug reaction; CI = confidence interval

### 

#### Incidence of ADRs

A total of 208 ADRs were reported from 188 patients; 170 patients experienced only one ADR, 16 patients experienced two ADRs, and two patients experienced three ADRs. The incidence rate of ADRs was 21.5%, while incidence of patients affected due to ADR was 19.5%. Of these 188 patients, 62 patients (33%) were admitted because of ADRs and 126 (67%) developed ADRs during their stay in the hospital. Therefore, the incidence of hospitalization due to ADRs was 6.4%, while the incidence of ARDs in hospitalized patients was 13%. On an average, there were 1.10 ADRs per patient.

Data showed preponderance of ADRs in female subjects as compared with males. Of 294 females enrolled, 82 (27.9%) suffered from one or the other form of ADR as compared with 106 of 672 males enrolled (15.8%). This difference was found to be statistically significant (Z = 4.37, *P* < 0.05). Incidence of ADRs was found to be higher in patients aged more than 65 years as compared with patients aged ≤65 years (24.9 *vs* 16.3%). This difference was also found to be statistically significant (Z = 2.52, *P* < 0.05).

There was a correlation between number of other comorbid conditions present in the patients and the incidence of ADR; it was 37.9% in patients with more than two comorbid conditions as compared with 10.7% in patients with ≤2 comorbid conditions, with a statistically significant difference (Z = 5.11, *P* < 0.05). Incidence of ADR was 23.5% in patients taking more than 10 drugs as compared with 8.4% in patients taking ≤10 drugs. This difference was statistically significant (Z = 5.27, *P* < 0.05).

Univariate analysis identified age, gender, number of comorbid conditions, and number of drugs being taken as risk factors for ADRs. Risk was twice as high in females as compared with males (OR = 2.07, CI = 1.47-2.91); 1.5 times as high as in patients more than 65 years age (OR = 1.5, CI = 1.08-2.09); six times higher in patients with more than two comorbid conditions as compared with patients with ≤2 comorbid conditions (OR = 6.16, CI = 4.30-8.83); and five times higher in patients taking more than 10 drugs as compared with patients taking ≤10 drugs (OR = 5.21, CI = 3.17-7.63). A logistic regression analysis showed that female gender (adjusted OR = 3.49) and elderly age group (adjusted OR = 0.53) were significant predictors of ADRs after controlling for the effect of other variables.

#### Type of ADRs

When categorized on the basis of Rawlins and Thompson classification for the type of ADRs, majority of ADRs were Type A (144, 69.23%), whereas Type B accounted for only 30.76% (64) of ADRs. The incidence of Type A ADRs was 14.9%, while the incidence of Type B ADRs was 6.6% of all enrolled patients. There was nonsignificant association between Type A or B reaction and patient’s characteristics, that is, age, gender, and number of comorbid conditions. Statistical analysis of the relationship between number of drugs and the type of reaction could not be applied.

#### Severity of ADRs

Majority of reactions, that is 134 (64.4%), were moderate, 46 (22.1%) were mild, and 28 (13.5%) were severe. The incidence of mild reactions was 4.7%, that of moderate reactions was 13.9%, and that of severe reactions was 2.8% of all enrolled patients. Mild and severe reactions were more common in males, whereas moderate reactions were significantly more common in females. Moderate reactions were common in both the age groups. Moderate and severe reactions were significantly more common in patients with more than two comorbid conditions (P<0.001). All types of ADRs were common in patients taking more than 10 drugs concurrently (*P*<0.001) [Table 2].

**Table 2:** Relationship between patient’s characteristics and severity of ADRs

*Characteristic*	*Severity (%)*			*χ^2^*	*P value*
	*Mild*	*Moderate*	*Severe*		
	*(n = 46)*	*(n = 134)*	*(n = 28)*		
Sex				
Male	34 (16.3)	64 (30.7)	20 (9.6)	12.39	0.002
Female	12 (5.8)	70 (33.6)	8 (3.8)		
Age (years)					
≤65	28 (13.4)	72 (34.6)	6 (2.9)	11.91	0.002
>65	18 (8.7)	62 (29.8)	22 (10.6)		
Comorbid condition				
≤2	22 (10.6)	50 (24.0)	2 (1.0)	13.06	0.001
>2	24 (11.5)	84 (40.3)	26 (12.5)		
Number of drugs				
≤10	12 (5.8)	10 (4.8)	0 (0)	16.39	0.0002
>10	34 (16.3)	124 (59.6)	28 (13.7)		

ADR = adverse drug reaction

#### Causality of ADRs

When analyzed on Naranjo ADR probability scale, majority of ADRs (118 of 208, 56.7%) were rated as probable followed by possible (90 of 208, 43.3%). Incidence of probable ADRs was 12.2% and that of possible reactions was 9.3% of all enrolled patients. The probable reactions were more common in males as compared with females; however, the difference was statistically nonsignificant. Probable reactions were common in patients with age less than 65 years, whereas possible reactions were significantly more common in patients aged more than 65 years. Moreover, both probable and possible reactions were common in patients with more than two comorbid conditions.

#### Drugs Involved and Commonly Reported ADRs

Of 208 reported ADRs in Cardiology department, cardiovascular drugs were responsible for maximum number of ADRs (138), followed by antibacterial agents (40) and intravenous contrast media (14). Among cardiovascular drugs, highest frequency was observed with nitrates (37), followed by diuretics (24). Most common adverse reaction reported was hypersensitivity skin reaction (54), followed by headache (37) and bradycardia (27) [Table 3].

**Table 3:** ADRs reported with causal drugs

*Drugs*	*Number of ADRs (%)*	*Reaction (No. of reports)*
Nitrates	37 (17.8)	Headache (37)
Diuretics	24 (11.5)	Hypokalemia (16)
		Hyperkalemia (8)
Combined CCBs and β blockers	21 (10.1)	Heart block (9)
		Bradycardia (12)
β blockers	19 (9.1)	Heart block (6)
		Bradycardia (13)
Amiodarone	16 (7.7)	Hypothyroidism (16)
Ceftriaxone	16 (7.7)	HS skin rxn (16)
Digoxin	15 (7.2)	Bradycardia (2)
		Arrhythmia (13)
Insulin	15 (7.2)	Hypoglycemia (15)
Contrast agents	14 (6.7)	HS skin rxn (14)
Ampicillin	13 (6.2)	HS skin rxn (13)
Metronidazole	7 (3.4)	HS skin rxn (7)
Streptokinase	6 (2.9)	Bleeding (6)
Piperacillin	4 (1.9)	HS skin rxn (4)
Theophylline	1 (0.5)	Cardiac arrest (1)

ADR = adverse drug reaction; CCBs= Calcium channel blockers; HS = Hypersensitive; rxn = reaction

#### Management and Outcomes

The offending drug was withdrawn in 99 (47.5%) reported cases, whereas 158 (75.9%) cases required an additional treatment for management of ADR. Dose was altered in 25 (12%) cases. Of all, 34 (16.3%) cases required no additional treatment or change in dose of offending drug. Total number of interventions done (316) was different from the total number of ADRs reported (208), because in many cases more than one intervention was done for management of a single ADR.

Of all the reported ADRs, 83 ADRs were serious in nature, that is, they either required or prolonged hospitalization or caused permanent disability or resulted in death. In the present study, 62 patients were admitted due to ADR, while ADRs prolonged hospital stay in three patients of bradycardia, 16 patients developed amiodarone-induced hypothyroidism, and two patients died due to development of ADR. Of 208 reported events of ADRs, 190 (91.3%) recovered fully, whereas 16 (7.7%) patients were still continuing with the medication for the ADR sequel, that is, hypothyroidism induced by amiodarone. Overall, only two reactions (0.96%) proved fatal. One patient died from theophylline-induced cardiac arrest and other had severe gastrointestinal bleeding after thrombolytic therapy with streptokinase.

## Discussion

An ADR is defined as ‘a response to a drug which is noxious and unintended and occurs at doses normally used in man for the prophylaxis, diagnosis or therapy of disease or for modification of physiological function.’[13] Growing importance of ADR monitoring can be gauged from the fact that Medical Council of India has recently recommended to establish Pharmacovigilance Committee in every teaching hospital.[14]

In the present study, of 966 patients enrolled, 62 (6.4%) were admitted to the hospital due to an ADR, while 126 (13%) patients developed ADRs during their stay in the hospital. In an earlier study, 1.2% hospital admissions were reported due to ADRs, while 11.24% patients developed ADRs during hospital stay, in cardiology set-up.[15] A higher incidence of ADRs reported in present study may be due to the fact that in present study two different methodologies were used to monitor ADRs. Moreover, in the present study, adverse effects of all the drugs prescribed in the cardiology department were studied in contrast to monitoring of adverse effects of only cardiovascular drugs in the earlier study.[15] Also, the incidence of ADRs may vary from place to place and even within a country because of difference in prescribing patterns.[16]

Predisposing factors like age, gender, comorbidity, number of drugs taken, and length of stay in the hospital have been reported as significant risk factors for the development of ADRs.[17 18] On multivariate analysis, the present study revealed that advanced age and female gender were the independent risk factors for development of ADRs.

Age is an important risk factor for ADRs, and incidence of ADRs increases steadily with age. This is due to pharmacodynamic and pharmacokinetic changes which, together with impairment of homeostatic mechanisms and the effect of coexisting disease, contribute to a significant increase in the incidence of ADRs. Another reason for increased incidence of ADRs in elderly is increased consumption of medicines.[19] Present study confirmed the fact that polypharmacy and comorbidity played a significant role in causation of ADRs in elderly patients, as 93% of the elderly patients (aged >65 years) were suffering from more than two diseases and all of them were receiving more than 10 drugs.

Earlier studies have also reported a higher incidence of ADRs in females.[20] The difference may be due to different rate of drug metabolism. Even after correction made for lean body mass and body surface area, significant gender difference do exist in drug metabolism.[21] Other major pharmacokinetic differences between the genders that can affect appearance of ADRs are lower body weight and glomerular filtration rate and a high percentage of body fat in women compared with men.[22]

In the present study, none of the reactions was categorized as certain because the present study was confined to patients admitted in intensive care unit; therefore, treatment interventions like rechallenge and dechallenge were not possible. It was observed that possible reactions were more commonly reported in elderly as compared with probable. The reason might be comorbidity leading to polypharmacy, as more drugs were being used, so an ADR cannot be attributed to a single drug. A high incidence of Type A reactions in comparison with Type B reactions (69.2% *vs* 30.7%) indicate that majority of ADRs were avoidable.

Although present study has some limitations like enrollment of only indoor patients, inability to assess outcome after dechallenge and rechallenge, inability to perform logistic regression on the number of drugs administered as a parameter (due to patient’s inability to recall exact numbers), this study would definitely give an insight into the pattern of ADRs in cardiology set-up in a tertiary care center and may help to increase awareness for further pharmacovigilance studies.
